# Postoperative wristwatch-induced compressive neuropathy of the hand: a case report

**DOI:** 10.1186/s13256-015-0625-5

**Published:** 2015-06-16

**Authors:** Laurence Weinberg, Manfred Spanger, Chong Tan, Mehrdad Nikfarjam

**Affiliations:** 1 Department of Anaesthesia, Austin Hospital, Heidelberg, VIC 3084 Australia; 2 Department of Surgery and Anaesthesia Perioperative Pain Unit, University of Melbourne, Heidelberg, 3084 VIC Australia; 3 Department of Radiology, Box Hill Hospital, Box Hill, 3128 VIC, Australia; 4 Department of Surgery, Austin Hospital, Heidelberg, VIC 3084 Australia

**Keywords:** Anaesthesia, Neuropathy, Neuropraxia

## Abstract

**Introduction:**

Postoperative peripheral nerve injuries are well-recognised complications of both surgery and anaesthesia and a leading cause of litigation claims. We present a rare cause of compressive sensory and motor neuropraxia of the median, ulnar and radial nerves of the right hand resulting from a wristwatch that was worn on the first postoperative night following minor surgery. Mechanisms of this compressive neuropathy are discussed, with specific recommendations made regarding the wearing of wristwatches, jewellery and constrictive clothing in the immediate postoperative period.

**Case presentation:**

A 12-year-old white boy presented with a complete glove and stocking sensory and motor neuropathy involving his right hand from a wristwatch that was worn on the first postoperative night following uneventful surgery for a minor procedure. Over the following 12 hours the oedema and erythema resolved with complete return of motor function. After 18 hours, the sensory deficit completely resolved.

**Conclusions:**

Postoperative neuropraxia is often preventable. Paediatric patients, especially if thin, may be particularly susceptible to a compression neuropathy from constrictive clothing or jewellery, in particular circumferential varieties such as wristwatches. These items should not be worn in the immediate postoperative period as pressure on peripheral nerves can result in severe and debilitating nerve injury. Education should be given to all medical staff, carers or parents of children undergoing surgery on the avoidance of wearing wristwatches, jewellery or constrictive clothing in the immediate postoperative period. Early medical evaluation of any postoperative nerve injury is of paramount importance.

## Introduction

Postoperative peripheral nerve injuries are well-recognised complications of both surgery and anaesthesia [1] and a leading cause of litigation claims against anaesthetists [2]. The mechanisms of these injuries are often multifactorial with common causes being direct nerve injury, nerve compression or stretch [3, 4] and nerve ischaemia [1]. We report a rare cause of postoperative compressive neuropathy of the right hand resulting from a wristwatch that was worn on the first postoperative night following uneventful surgery.

## Case presentation

A fit and healthy 12-year-old white boy (height 150cm, weight 40kg) underwent elective removal of a 2×2cm benign skin lesion from his anterior abdominal wall. He had no past medical history and was not on any medications. His physical examination preoperatively was normal. Surgery was scheduled for the late afternoon. He was fasted for 6h for solids and 2h for clear fluids. The World Health Organization surgical checklist for safety in the operating room [5] was complied with intraoperatively, which included the prohibition of jewellery and other constrictive clothing items worn during surgery. After establishing intravenous access with a 22-guage cannula in a vein in the cubital fossa of his left arm, anaesthesia was induced with propofol (140mg) and fentanyl (50ug). A size 3 classic laryngeal mask was inserted for airway management, and spontaneous ventilation was maintained with 1 minimum alveolar concentration sevoflurane in a 50% oxygen to air mixture. Five millilitres of a local anaesthesia solution (lignocaine 2% with 1:200,000 adrenalin) was injected into the surgical area for local anaesthesia and postoperative analgesia. During surgery both of his arms were positioned by his side in a neutral position, supported by foam padding on the operating table to minimise pressure. The abdominal lesion was removed uneventfully and the procedure took 22min. Continuous electrocardiography, pulse saturation monitoring, and intermittent blood pressure measurements every 3min were normal. After uneventful emergence from anaesthesia, the laryngeal mask was removed, and he was transferred to the postoperative recovery unit for a 30-min observation period. He was haemodynamically stable, with normal arousal to voice and command. He required no further analgesia and was transferred to the ward at 1830 hours. On the ward his observations remained stable. After a light meal and when completely conversant and cooperative, he was discharged home under parental supervision.

**Fig. 1:**
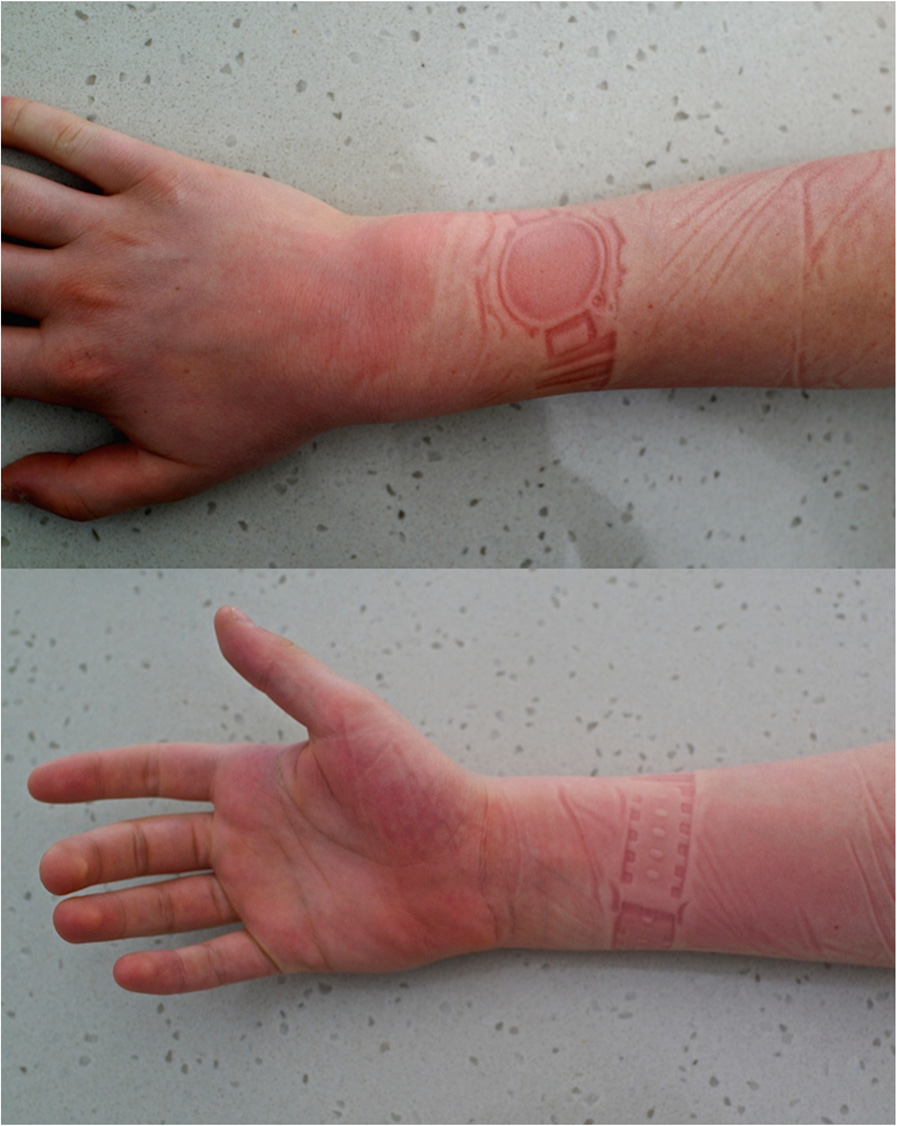
Dorsal and volar surfaces of the right hand demonstrating a wristwatch imprint from the face and the strap of the watch with resulting erythema and swelling

**Fig. 2:**
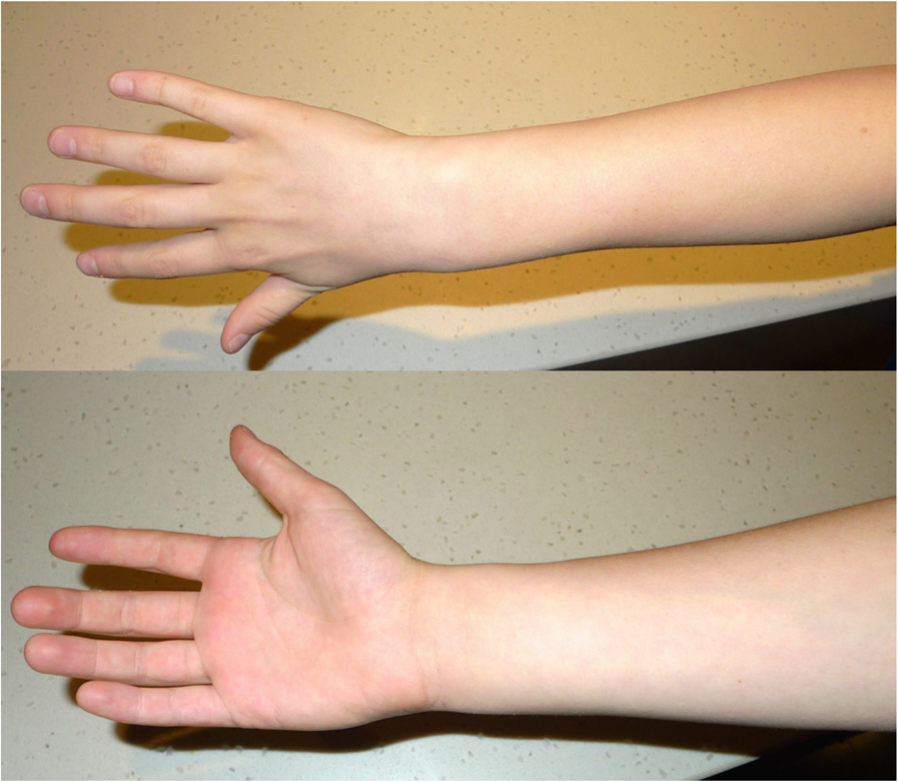
Dorsal and volar surfaces of the right hand taken 48h later with complete resolution of the erythema and oedema

He presented with his parents to the emergency department the following morning complaining of severe pain, paraesthesia and weakness of the right hand. A clinical examination revealed a circumferential wristwatch pressure imprint over his anterior and posterior distal right forearm (Figs. 1 and 2), as a result of sleeping with his wristwatch on. His hand was erythematous, swollen and painful to touch. There were complete sensory and motor deficits involving his right hand in a glove and stocking distribution from the wristwatch pressure imprint distally, including involvement of all five digits. A neurological examination showed inability to differentiate cooling and heat, sharp and dull, and there was a deficit in 2-point discrimination in the radial, median and ulnar nerve root distributions, most severe in the territory of the superficial cutaneous branch of the radial nerve. In addition there was complete motor deficit in the median, radial and ulnar nerve distributions.

He was admitted to the overnight short stay unit where his hand was elevated. A non-steroidal anti-inflammatory analgesic (ibuprofen 400mg) was prescribed 8 hourly for analgesia. Movement of his left hand was encouraged, and over the following 12h the oedema and erythema resolved completely, with complete return of motor function. After 18h, the sensory deficit had also completely recovered. In view of the rapid neurological recovery, quantitative nerve function assessments, for example nerve conduction studies, were not performed. He was discharged home and reviewed 24h later at the surgical out-patients clinic where his hand function examination remained normal.

## Discussion

Compression neuropathy to the superficial cutaneous branch of the radial nerve was originally described by Wartenberg in 1932 [6]; however, to the best of our knowledge this is the first reported case of a postoperative local compressive neuropathy of the hand secondary to a wristwatch compression injury. Although the aetiology and pathophysiological mechanisms of intraoperative neuropraxia are well described, isolated postoperative compression neuropraxia is uncommon. Intraoperatively, both surgical and anaesthesia factors have been reported to predispose to nerve injuries [1]. Nerves may be unduly susceptible to injury as a result of a pre-existing peripheral neuropathy or hereditary predisposition, or as a result of hypovolaemia, dehydration, hypotension, hypoxia and electrolyte disturbances [1]. None of these factors were present in the case described.

**Fig. 3:**
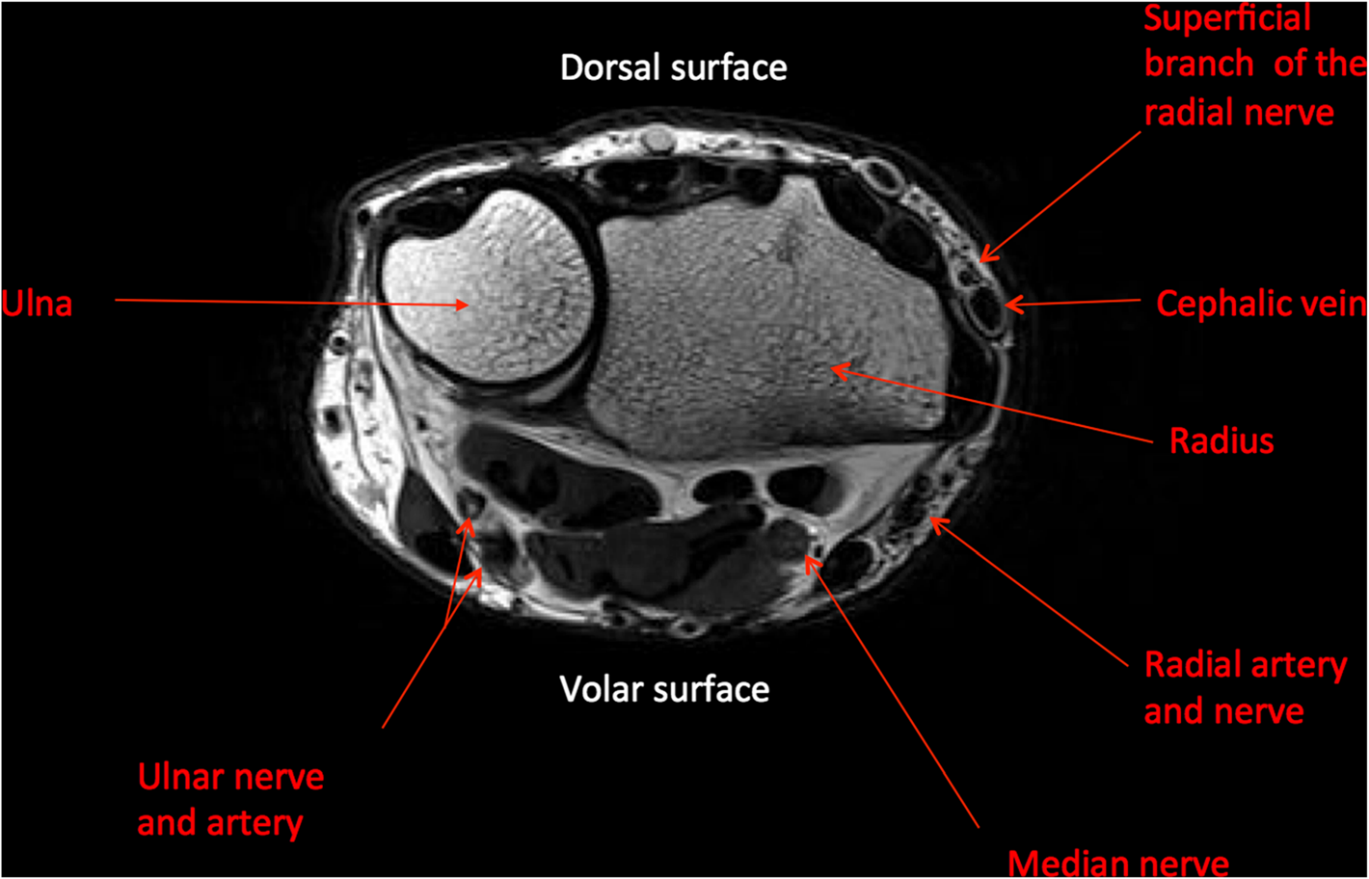
Wrist magnetic resonance imaging anatomy. T1-weighted axial view demonstrating the specific proximity of the ulnar, radial and median nerves to the skin

Superficial nerves, such as the ulnar, radial, sciatic and common peroneal nerves, have been reported to be particularly vulnerable to perioperative injury, especially in patients who are thin [7]. In the case described here, the most likely mechanism of the neuropathy was the direct compression of the nerves of the hand from the wristwatch, which occurred during sleep. The circumferential nature of this compression injury also compromised venous return, causing interstitial oedema, swelling and erythema, exacerbating the compression neuropathy. The severity of the neuropraxia may also have been exacerbated by the fact that the patient was a child, with thin extremities. From the diagram presented in Fig. 3, it can be appreciated how superficial the ulnar, median and radial nerve are in relation to the skin, therefore any prolonged compression, as described in this case, could explain the mechanism of the compression neuropathy.

It is possible that the residual effects of both anaesthesia agents and analgesics administered during the treatment resulted in a more prolonged and deeper sleep than normal. In addition, direct compression of the hand against the child’s body during the night could have further compounded the venous congestion. Other possible contributory factors could be compression of the ulnar and radial nerve against the hard operating table and the humerus during the time of the surgical procedure; however, this is unlikely as the child did not complain of any discomfort of the hand on discharge. Intraoperative ulnar and radial nerve injuries may be more common if the forearm is fully extended and in the pronated position, or if the nerve is stretched around the medial epicondyle during extreme flexion of the elbow across the chest [1, 2], or if patient is in the lateral position and the uppermost arm is abducted beyond 90 degrees and suspended from a vertical screen support [8]; however, we consider these factors unlikely to be contributory as the operation was of very short duration, and his arm was positioned during the operation in a neutral position by his side, with a soft gelfoam pressure mattress as part of the operating bed structure.

Detailed attention to intraoperative and postoperative factors is paramount in the prevention of perioperative peripheral nerve injuries. Any perioperative peripheral nerve injury that has not fully resolved within 24h should be urgently referred to a neurologist for further neurophysiological evaluation and investigations, which can provide useful diagnostic and prognostic information [1]. Detailed anaesthetic documentation of intraoperative limb positioning, in addition to protective measures taken to prevent peripheral nerve injuries should be standard for all cases. Although careful intraoperative positioning and adequate padding can reduce and prevent intraoperative nerve injury, postoperative factors are equally important. Constrictive clothing and jewellery, particularly those of a circumferential nature such as wristwatches, should not be worn in the immediate postoperative period, as these can result in venous congestion and a compressive neuropathy if slept on, which may be masked by the residual and sedating effects of the anaesthetic. As demonstrated from this report, paediatric patients may be particularly susceptible. Adequate verbal and written instruction to the parents on the avoidance of wearing wristwatches and jewellery in the immediate postoperative period should form part of a standard anaesthetic and surgical care plan.

## Conclusions

Postoperative neuropraxia is often preventable. Paediatric patients, especially if thin, may be particularly susceptible to a compression neuropathy. Constrictive clothing and jewellery, in particular circumferential varieties such as wristwatches, should not be worn in the immediate postoperative period, as pressure from these items on peripheral nerves can result in severe and debilitating nerve injury. Education should be given to all carers or parents of children undergoing anaesthesia to avoid wearing such items in the immediate postoperative period. Early medical evaluation of any postoperative nerve injury is of paramount importance.

## Consent

Written informed consent was obtained from the patient’s legal guardian for publication of this case report and any accompanying images. A copy of the written consent is available for review by the Editor-in-Chief of this journal.
